# miR‐3133 inhibits gastrointestinal cancer progression through activation of Hippo and p53 signalling pathways via multi‐targets

**DOI:** 10.1111/jcmm.17880

**Published:** 2023-08-09

**Authors:** Ling Zhou, Hui Guo, Quan Liao, Jianping Zou, Yi Le, Ziling Fang, Jianping Xiong, Shanshan Huang, Jun Deng, Xiaojun Xiang

**Affiliations:** ^1^ Department of Oncology The First Affiliated Hospital of Nanchang University Nanchang China; ^2^ Jiangxi Key Laboratory for Individualized Cancer Therapy Nanchang China

**Keywords:** gastrointestinal cancer, hippo signalling pathway, miR‐3133, p53 signalling pathway, RNF146

## Abstract

**Background:**

Malignant cell growth and chemoresistance, the main obstacles in treating gastrointestinal cancer (GIC), rely on the Hippo and p53 signalling pathways. However, the upstream regulatory mechanisms of these pathways remain complex and poorly understood.

**Methods:**

Immunohistochemistry (IHC), western blot and RT‐qPCR were used to analyse the expression of RNF146, miR‐3133 and key components of Hippo and p53 pathway. CCK‐8, colony formation, drug sensitivity assays and murine xenograft models were used to investigate the effect of RNF146 and miR‐3133 in GIC. Further exploration of the upstream regulatory mechanism was performed using bioinformatics analysis, dual‐luciferase reporter gene, immunoprecipitation assays and bisulfite sequencing PCR (BSP).

**Results:**

Clinical samples, in vitro and in vivo experiments demonstrated that RNF146 exerts oncogenic effects in GIC by regulating the Hippo pathway. Bioinformatics analysis identified a novel miRNA, miR‐3133, as an upstream regulatory factor of RNF146. fluorescence in situ hybridization and RT‐qPCR assays revealed that miR‐3133 was less expressed in gastrointestinal tumour tissues and was associated with adverse pathological features. Functional assays and animal models showed that miR‐3133 promoted the proliferation and chemotherapy sensitivity of GIC cells. miR‐3133 affected YAP1 protein expression by targeting RNF146, AGK and CUL4A, thus activating the Hippo pathway. miR‐3133 inhibited p53 protein degradation and extended p53's half‐life by targeting USP15, SPIN1. BSP experiments confirmed that miR‐3133 promoter methylation is an important reason for its low expression.

**Conclusion:**

miR‐3133 inhibits GIC progression by activating the Hippo and p53 signalling pathways via multi‐targets, including RNF146, thereby providing prognostic factors and valuable potential therapeutic targets for GIC.

## 1 INTRODUCTION

Gastric cancer and colorectal cancer are among the most prevalent and deadliest gastrointestinal cancer (GIC), contributing to substantial morbidity and mortality rates.^1^ The complex pathogenesis of GIC involves intricate interactions between genetic, epigenetic and environmental factors, leading to the dysregulation of multiple signalling pathways and cellular processes.^2^, ^3^ Understanding the molecular mechanisms underlying GIC is paramount for the development of effective diagnostic tools, prognostic markers and targeted therapies.

The Hippo and p53 signalling pathways play an important roles in physiological and pathological processes.^4^, ^5^ The Hippo signalling pathway is highly conserved in mammals and regulates organ size and tumorigenesis.^6^, ^7^ Its key components include a kinase cascade, mammalian ste20‐like protein kinase 1/2 (MST1/2), a large tumour suppressor kinase 1/2 (LATS1/2) and two transcription coactivators, Yes‐associated protein (YAP) and Tafazzin (TAZ).^8^ When the Hippo signalling pathway is activated, MST1/2‐mediated phosphorylation and activation of LATS1/2 further phosphorylates and inactivates YAP/TAZ, causing its retention in the cytoplasm and degradation. In contrast, inactivation of the Hippo pathway leads to YAP/TAZ translocation to the nucleus and combination with transcription factors, such as TEADs. This promotes tumorigenesis via the activation of target genes, such as CTGF and CYR61.^9^, ^10^ p53, an anticancer gene, causes cell cycle arrest, apoptosis and senescence by manipulating downstream targets.^11^, ^12^ Degradation of the p53 protein leads to the inactivation of the p53 signalling pathway and tumour development.^13^, ^14^ MDM2 is the most widely studied ubiquitination mechanism of p53.^15^ As an E3 ubiquitin ligase, MDM2 targets p53 for proteasome degradation.^16^ In addition, it inhibits p53 transcription factor function by promoting the nuclear export of p53.^17^ p53 protein can be regulated by CUL4A, USP15, SPIN1 and many other molecules through MDM2, thus affecting the malignant phenotype of tumours.^14^, ^18^, ^19^, ^20^ Despite a considerable amount of research effort, the upstream regulatory mechanisms of the p53 and Hippo signalling pathways require further investigation.

As an E3 ubiquitin ligase, RING finger protein 146 (RNF146) has a PAR‐binding motif in the Trp‐Trp‐Glu (WWE) and RING domains, which can recognize poly (ADP‐ribose) (PAR) and target them for proteasomal degradation.^21^ RNF146 is involved in several physiological and pathological processes such as bone dynamics, energy metabolism and oxidative stress.^22^, ^23^ Furthermore, RNF146 also participates in tumorigenesis and progression via multiple pathways which include regulating the Wnt/β‐catenin pathway to promote the biological behaviour of colorectal cancer and non‐small cell lung cancer, upregulating PD‐L1 expression to induce immune escape of hepatocellular carcinoma.^24^, ^25^, ^26^ However, the role of RNF146 in GIC, its interactions with other pathways, and upstream regulatory mechanisms remain unclear.

By simultaneously repressing a variety of target genes through binding to their 3′‐untranslated regions (3′‐UTRs), microRNAs (miRNAs) can potentially affect multiple steps in cancer progression and tumorigenesis.^27^ MicroRNA‐3133 (miR‐3133), a novel miRNA, is located at the common fragile site (CFSs) and is defined as a cancer‐related cytogenetic miRNA.^28^ Thus far, only five studies have reported the pathophysiological effects of miR‐3133, and only two of which are tumour‐related. These included retinoblastoma and clear cell renal cell carcinoma, in which miR‐3133 plays a suppressive role in tumour progression. As such, the role of miR‐3133 in tumours requires further investigation.

In this study, we primarily explored the upstream regulatory mechanisms of the Hippo and p53 pathways. First, we performed a series of experiments that analysed the role of RNF146 in GIC and its effect on the Hippo pathway. Second, we investigated the effect of miR‐3133 on multiple target molecules including RNF146 and the role of miR‐3133 in regulating the Hippo and p53 pathways. Finally, the expression and epigenetic status of miR‐3133 in GIC were explored. Our findings aim to clarify whether miR‐3133 can act as a promising prognostic factor and valuable therapeutic target for GIC.

## 2 MATERIALS AND METHODS

### 2.1 Tissue collection and ethics statement

A total of 160 formalin‐fixed paraffin‐embedded cancer samples, along with complete clinical and pathological data, were collected from gastric cancer (GC) patients who underwent surgical resection at the First Affiliated Hospital of Nanchang University, Nanchang, China, between 2016 and 2018. The clinicopathological features of the patients are provided in Table 1. Another 20 pairs of fresh GC tissues and 42 pairs of fresh colorectal cancer (CRC) tissues and their matched noncancerous tissues were obtained from the general surgery room between 2018 and 2021, immediately frozen, and stored in liquid nitrogen for subsequent experimentation. The protocols used in this study were approved by the ethics committee of the First Affiliated Hospital of Nanchang University. Written informed consent was obtained from all the patients.

**TABLE 1:** Clinicopathological characteristics and tumour expression of RNF146 in gastric cancer patients.

Patient characteristics	*N*	RNF146 expression (%)		*p*‐Value
		Low or None	High	
Age (years)	160	63 (39.4)	97 (60.6)	
<65	86	30 (34.9)	56 (65.1)	0.210
≥65	74	33 (44.6)	41 (55.4)	
Sex				
Male	88	34 (38.6)	54 (61.4)	0.833
Female	72	29 (40.3)	43 (59.7)	
Tumour size (cm)				
<5	88	41 (46.6)	47 (53.4)	**0.039**
≥5	72	22 (30.6)	50 (69.4)	
Differentiation				
Moderately or well	92	39 (42.4)	53 (57.6)	0.364
Poorly	68	24 (35.3)	44 (64.7)	
Depth of invasion				
T_1_–T_2_	47	27 (57.4)	20 (42.6)	**0.003**
T_3_–T_4_	113	36 (31.9)	77 (68.1)	
Lymph node metastasis				
Without	72	34 (47.2)	38 (52.8)	0.066
With	88	29 (33.0)	59 (67.0)	
TNM stage				
I‐II	79	37 (46.8)	42 (53.2)	0.056
III‐IV	81	26 (32.1)	55 (67.9)	
Lauren classification				
Intestinal‐type	83	33 (39.8)	50 (60.2)	0.918
Diffuse‐type	77	30 (39.0)	47 (61.0)	

*Note*: All the factors were analysed by the chi‐square test. Entries in bold font indicate statistically significant (*p* < 0.05).

### 2.2 Cell culture

Human gastric and colorectal cancer cell lines (AGS, BGC‐823, MGC‐803, SGC‐7901, MKN‐45, HGC‐27, HCT116 p53^+/+^ and HCT116 p53^−/−^) and human gastric epithelial cell line GES‐1 were purchased from the American Type Culture Collection (ATCC) and Shanghai Institute of Cell Biology (Shanghai, China). The cells were maintained in Roswell Park memorial institute (RPMI)‐1640 or Dulbecco's modified eagle medium (DMEM) medium (both from HyClone) supplemented with 10% fetal bovine serum (FBS, Biological Industries) in an atmosphere containing 5% CO_2_ at 37°C.

### 2.3 RNA extraction and RT‐qPCR analyses

Total RNA was extracted from GC tissues, CRC tissues and paired adjacent noncancerous tissues. Further complementary DNA (cDNA) was obtained and Real‐time PCR was performed as previous described.^29^ GAPDH served as an internal control. Relative mRNA expression of the indicated genes was calculated using the 2^−ΔΔCT^ method. The PCR primer sequences used are listed in Table S1.

### 2.4 Cell infection and transfection

RNF146 overexpression plasmid, RNF146 short hairpin RNAs (shRNAs), miR‐3133 mimics and inhibitors, and their corresponding controls were introduced into cancer cells using Turbofect Reagent (Thermo Fisher, St Louis, MO, USA) according to the manufacturer's instructions. All shRNAs and miRNA mimic/inhibitor constructs were designed and purchased from GenePharma (Suzhou, China). The sequence of the human miR‐3133 inhibitor was 5′‐AUUGGGUUUUAAGAGUUCUUUA‐3′. The miR‐3133 mimics sequence was 5′‐UAAAGAACUCUUAAAACCCAAU‐3′ and the NC sequence was 5′‐ ACGUGACACGUUCGGAGAATT −3′. The RNF146 shRNA sequences were as follows: RNF146 shRNA #1, 5’‐GTGACACCAATACTGTAAAT‐3′; RNF146 shRNA #2, 5′‐ CTAGTGTCTTCTGTAAGGC‐3′. The amplified human RNF146 sequences were subcloned into a pcDNA3.1 vector.

### 2.5 Western blotting

Protein samples were lysed in ice‐cold radioimmunoprecipitation assay buffer (Beyotime, Shanghai, China). The lysates were separated using 10% SDS‐PAGE and then transferred onto a nitrocellulose membrane (Millipore). The nitrocellulose membrane was blocked and incubated with the corresponding primary antibodies at 4°C overnight. Afterwards, the membrane was washed thrice with TBST and incubated with goat anti‐rabbit/mouse IgG (H + L) HRP secondary antibody (ZB‐2305; ZB‐2301; Zhongshan Golden Bridge, Beijing, China) at room temperature for 1 h. Finally, the proteins of interest were detected using an ECL reagent (WBULS0500; Merck Millipore, Darmstadt, Germany). The primary antibodies used are listed in Table S2.

### 2.6 5‐ethynyl‐2′‐deoxyuridine assay

5‐ethynyl‐2′‐deoxyuridine (EdU) assay was performed using the EdU Apollo‐567 kit (C10310‐1, RiboBio). Briefly, HGC‐27 cells and HCT‐116 cells were seeded into 96‐well plates (1 × 104 cells/well) and cultured overnight. EdU (10 μM) was added into each well and incubated for 3 h at 37°C. After the cells were fixed with 4% polyoxymethylene at 25°C for 30 min and decolorized with glycine (2 mg/mL) at 25°C for 5 min, the cell nuclei were stained with Hoechst 33342 at 25°C for 30 min. The fluorescence of cells was captured with a Zeiss LSM 510 inverted fluorescence microscope (Zeiss). The EdU ratio was calculated as follows: Number of EdU‐positive cells/number of Hoechst 33342‐positive cells ×100%.

### 2.7 CCK‐8 assay and colony formation assay

To assess cell viability, post‐transfected cells were seeded in 96‐well plates at 10^3^ cells per well and cultured for indicated days. The CCK‐8 assay was performed according to the manufacturer's instructions. Optical density values were measured at 450 nm absorbance using a microplate reader (Molecular Device, SpecrtraMax M5e). For the colony formation assay, GC or CRC cells (1000 per well) were seeded in 6‐well plates 36 h post‐transfection and cultured in RPMI ‐1640 medium containing 10% fetal bovine serum for 2 weeks. The cells were then fixed with 75% ethanol and stained with a 0.1% crystal violet solution. Only colonies with more than 50 cells per colony were counted. Experiments were performed in triplicate.

### 2.8 Dual‐luciferase reporter gene assay

Both the wild‐type 3′‐UTR sequence, containing an established complementary region, and the mutant 3′‐UTR sequence, lacking the mutant complementary region, of the indicated genes were cloned into a pGL3 luciferase reporter vector (Invitrogen). Additionally, both wild‐type miR‐3133 (3′‐UAACCCAAAAUUCUCAAGAAAU‐5′) and mutated miR‐3133 (3′‐UAACCCAAAAUUCUCUUCAAAU‐5′) were designed for use in luciferase reporter gene assay. Cells were harvested 48 h after transfection and luciferase activity was detected using the Dual‐luciferase Reporter Assay Kit (Promega, E1910) according to the manufacturer's instructions. All experiments were repeated at least thrice.

### 2.9 In vivo animal study

Female BALB/c mice (4 weeks old) were obtained from Zhejiang Weitong Lihua Laboratory Animal Technology Co., Ltd. (Zhejiang, China) and randomly grouped. 5 × 10^6^ GC cells stably transfected with lentivirus shRNF146‐1, lentivirus miR‐3133 or their controls were injected subcutaneously into the right armpit of nude mice (*n* = 6 per group). The tumour volumes were determined at the indicated time points (volume [mm^3^] = [length × width^2^]/2). Approximately 1 month after injection, nude mice were euthanized, and tumours were peeled off, weighed and photographed. This xenograft study was approved by the Ethics Committee of the First Affiliated Hospital of Nanchang University.

### 2.10 Analysis of protein stability

Cells were transfected, incubated with cycloheximide (50 μg/ml) and harvested at different time points (0, 15, 30, 45, 60 and 90 min). Subsequently, the levels of various proteins were analysed by western blot analysis and quantified using ImageJ software.

### 2.11 Immunoprecipitation assays

An immunoprecipitation assay was performed to detect p53 ubiquitination. HCT116 p53^−/−^ cells were seeded into 10 cm dishes and transfected with miR‐3133 inhibitors, miR‐3133 mimics, HA‐MDM2, His‐Ub or p53 plasmid (10 μg each) for specific needs. The plasmids encoding HA‐MDM2, p53 and His‐Ub have been previously described.^20^ After 36 h, the cells were treated with MG132 (10 μM) for 6 h to inhibit proteasomal degradation and then harvested for cell extracts. Cell lysates were then immunoprecipitated with anti‐p53 antibodies and protein A/G beads (#22202–20, Beaver Nano‐Technologies) at 4°C overnight. The levels of ubiquitination of p53 were determined using an anti‐Ub antibody by immunoblotting.

### 2.12 Bisulfite sequencing PCR

The methylation status of miR‐3133 promoter CpG islands in tissues was analysed using bisulfite sequencing PCR (BSP). According to the manufacturer's instructions, DNA was extracted from GC and adjacent tissues using a DNA extraction kit (Tiangen Biotech, DP304) and treated with sodium bisulfite using the Methylation‐Gold Kit (D5005S (10), ZymoResearch). The treated DNA was then amplified by PCR using appropriate MSP primers. The primers were designed using MethPrimer (http://www.urogene.org/methprimer), and the sequences were as follows: F: TTGTTATTAGGTTGGGGTGTAGTGT and R: ACATAAATCCACACAAAACTTATACATAAAC. The PCR conditions were as follows: 95°C for 5 min; 34 cycles of 95°C for 30 s, 55°C for 30 s and 72°C for 30 s, with a final extension step at 72°C for 5 min. The PCR‐amplified products were ligated to T‐Vector PMD19 (6013, Takara Biotechnology, Dalian, China) and transformed into *Escherichia coli* DH5α cells. LB agar plates containing kanamycin (50 μg/mL) were used to select qualified colonies at 37°C temperature overnight. Colonies were selected and cultured in LB^amp+^ medium overnight, and the obtained plasmids were sequenced using ABI 3730XL (ABI).

### 2.13 Immunohistochemistry staining and immunofluorescence

Immunohistochemical staining was performed as previously described.^30^ Staining results were evaluated by two pathologists based on the proportion of positively stained cells and staining intensity. The proportion was scored as 0 (0%), 1 (0%–10%), 2 (10%–50%) and 3 (50%–100%). The staining intensity was scored as 0 (negative), 1 (weak), 2 (moderate) and 3 (strong). The final protein expression scores were calculated by multiplying these two scores. Patients with an overall score <4 were considered to have low expression, and those with an overall score ≥4 were considered to have high expression of RNF146, CUL4A, AGK, YAP1, TEAD1 and USP15. After 48 h transfection, Yap protein levels were detected by immunofluorescence assay as previous described.^31^

### 2.14 RNA fluorescence in situ hybridization

Fresh GIC tissues were fixed with paraformaldehyde immediately after excision, and then 4 μm sections were prepared after dehydration and paraffin embedding. The sections were then dewaxed, dehydrated, digested with proteinase K, prehybridized and examined with an oligonucleotide‐modified probe sequence (ATTGGGTTTTAAGAGTTCTTTA) for human miR‐3133.

### 2.15 Bioinformatics website

We assessed genomic and mRNA changes involving RNF146 in human cancers using the online database Cbioportal (http://www.cbioportal.org). Survival analysis of patients was performed using Kaplan–Meier plotter website data (https://kmplot.com/analysis/). Based on bioinformatics tools (TargetScan [www.targetscan.org] and miRanda [www.microrna.org]), we speculated that miR‐3133 is a putative miRNA that regulates RNF146 expression. Bioinformatic analysis tools MethPrimer (http://www.urogene.org/methprimer2/) and NCBI were used to identify miR‐3133 CpG islands, and miRPath (http://www.microrna.gr/miRPathv3/) was used for GO and KEGG analyses of miR‐3133 biological functions.

### 2.16 Statistical analysis

All experiments were performed independently thrice, and all data are presented as mean ± SEM. Statistical analysis was performed using SPSS v.22.0 (SPSS, Chicago, IL, USA). Quantitative data are presented as the mean ± standard deviation. Student's *t*‐test or chi‐square test was used to analyse the statistical differences between the two groups. Survival curves were plotted using the Kaplan–Meier method and analysed using the log‐rank test. Statistical significance was set at *p* < 0.05.

## 3 RESULTS

### 3.1 RNF146 acts as an oncogene in GIC

**FIGURE 1:**
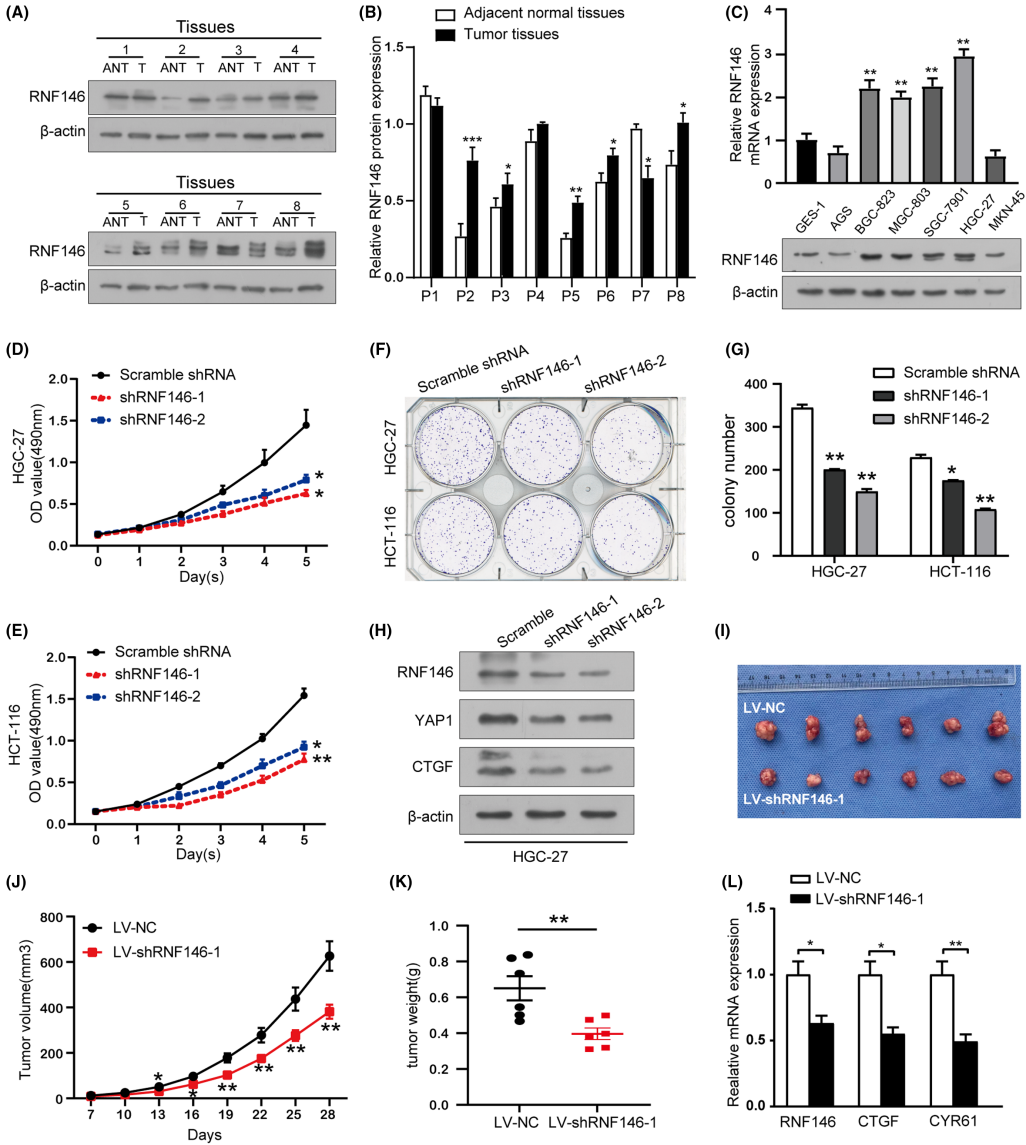
RNF146 acts as an oncogene in gastrointestinal cancer (GIC). (A) and (B) Western blot images and quantification of RNF146 in gastric cancer (GC) tissues (T) and adjacent normal tissues. (C) The protein and mRNA levels of RNF146 in GC cells and normal epithelial mucosal cell lines GES‐1 detected by western blotting and RT‐qPCR. (D)–(G) Proliferation of GIC cells examined by CCK‐8 assay and colony formation assay after transfection with RNF146 short hairpin RNAs (shRNAs) or the negative control. (H) YAP1 and its downstream molecule, CTGF, were detected after the downregulation of RNF146. (I)–(L) Xenograft tumour models derived from HGC‐27 cells expressing LV‐scramble shRNA (LV‐NC) or LV‐shRNF146. (I) Images of xenograft tumours harvested at the end of the experiments. (J) Growth curves of xenograft tumours. (K) Comparison of the average weights of tumours collected from the above two groups. (L) RT‐qPCR of RNF146, CTGF and YAP1 in xenograft tumours (*p < 0.05, **p < 0.01 vs. corresponding control groups).

To explore the role and expression of RNF146 in GIC, a series of biochemical experiments was conducted. Previous studies have shown that RNF146 is highly expressed and associated with poor prognosis in CRC.^26^ Here, we focused on exploring the role of RNF146 in GC. Western blot assays showed that RNF146 protein expression increased in human GC tissue samples paired with adjacent non‐tumour tissues (5/8, 62.5%, Figure 1A,B) as well as in most GC cell lines compared to that in the gastric normal epithelial mucosa cell line GES‐1 (Figure 1C). In line with the above results, immunohistochemical staining of RNF146 was deeper in tumour tissues than in normal tissues and was positively correlated with the TNM stage (Figure S1A). In addition, the relationship between the expression of RNF146 and the clinicopathological picture of patients was explored. As shown in Table 1; Figure S1B,C, RNF146 overexpression was significantly associated with tumour size (*p* = 0.039), depth of invasion (*p* = 0.003) and poor overall survival (median overall survival: 22.10 vs. 31.35 months, *p* = 0.0216) in GC patients. Thus, previous studies and our findings suggest that RNF146 is overexpressed in GIC and is associated with unfavourable clinical outcomes and poor survival of patients.

The oncogenic effect of RNF146 was further investigated in vivo and in vitro. We transfected Scramble shRNA and RNF146 shRNA into HGC‐27 and HCT‐116 cells, respectively, and verified the transfection efficiency using immunoblotting and RT‐qPCR (Figure S1D,E). As expected, both the CCK‐8 and colony formation assays indicated that the downregulation of RNF146 suppressed cell proliferation in HGC‐27 and HCT‐116 cells (Figure 1D–G). In vivo experiments were performed by injecting RNF146‐knockdown HGC‐27 cells into the armpit of BALB/C‐nude mice, and the xenograft tumours were measured for 28 days. The results showed that the average xenograft tumour weight and tumour volume derived from HGC‐27 cells transduced with lentivirus shRNF146‐1 were significantly decreased compared to those of the respective negative control (Figure 1I–K). Moreover, previous studies have shown that RNF146 is a critical upstream regulator of YAP1.^32^ To verify this, we conducted RT‐qPCR and western blot assays in xenograft tumours and GC cells. The results revealed that the expression of YAP1 and its downstream target genes was markedly decreased in RNF146‐knockdown HGC‐27 cells (Figure 1H; Figure S1F) and xenograft tumours (Figure 1L). Additionally, the knockdown efficiency of RNF146 in the xenograft tumours was further confirmed after the excision of the tumour mass (Figure S1G). Collectively, these data support that RNF146 acts as an oncogene in GIC.

### 3.2 RNF146 is a direct target of miR‐3133

**FIGURE 2:**
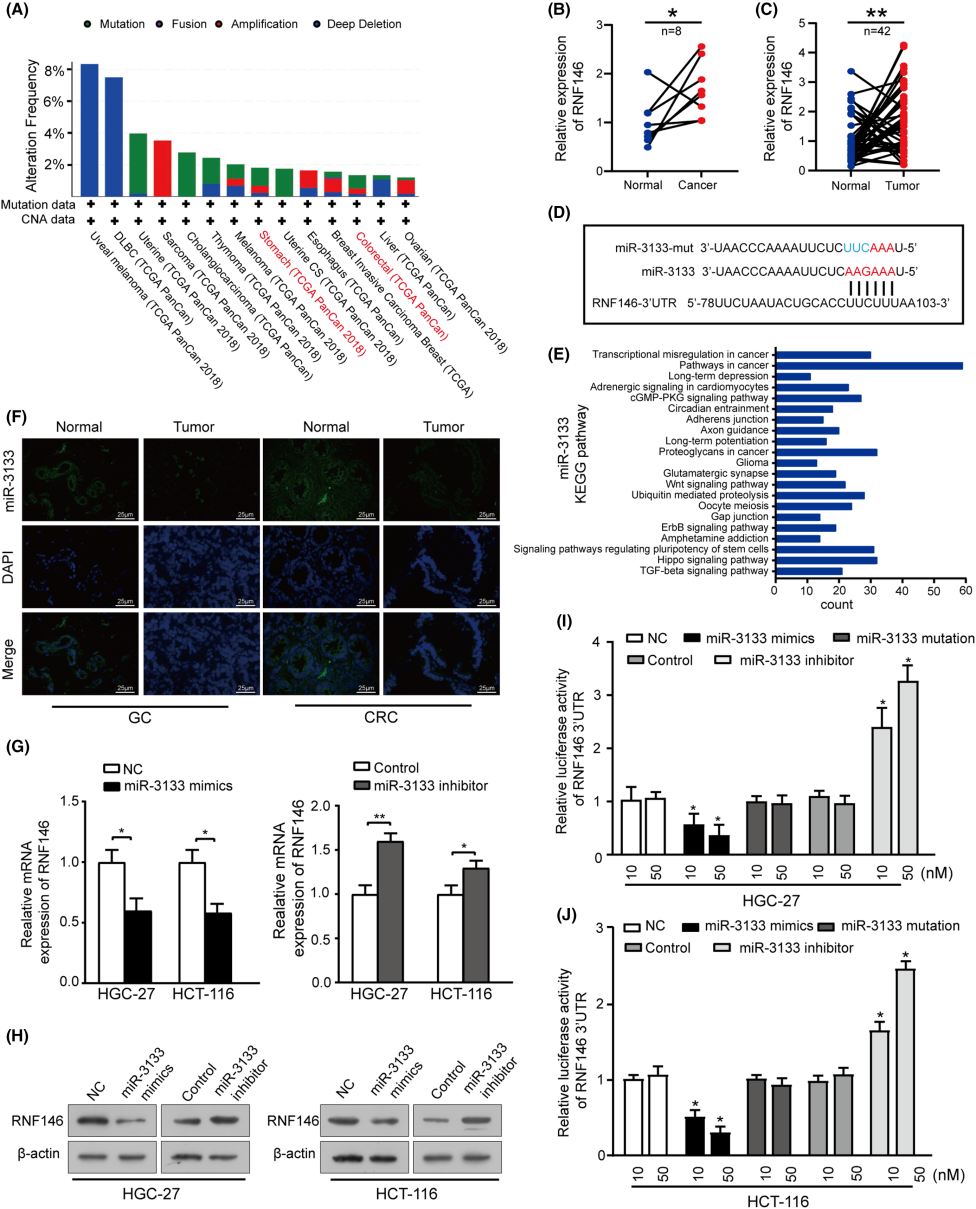
RNF146 is a direct target of miR‐3133. (A) The alteration frequency of RNF146 in various cancer tissues obtained from the cBioPortal for Cancer Genomics website (http://www.cbioportal.org/) derived from the TCGA database. (B) and (C) Relative RNF146 mRNA expression in eight pairs of fresh gastric cancer (GC) tissues and 42 pairs of fresh colorectal cancer (CRC) tissues. (D) Schematic diagram of the putative binding sites of miRNA‐3133 in the RNF146 3′‐UTR (red) and mutated binding sites of miRNA‐3133 (blue). (E) KEGG pathway analysis of miRNA‐3133 in miRPath (http://www.microrna.gr/miRPathv3/). (F) Fluorescence in situ hybridization analysis of miRNA‐3133 in GC and normal tissues, as well as in CRC and normal tissues. (G) and (H) RT‐qPCR and Western blot analyses of RNF146 expression in HGC‐27 and HCT‐116 cells after transfection with miR‐3133 mimics, miR‐3133 inhibitor or negative control. (I) and (J) Luciferase activity of RNF146 3′‐UTR in HGC‐27 and HCT‐116 cells transfected with wild‐type miR‐3133, mutant miR‐3133, miR‐3133 inhibitor or negative control (*p < 0.05, **p < 0.01 vs. corresponding control groups).

To explore the upstream regulatory mechanism of RNF146, we first assessed genomic changes involving RNF146 in human cancers using the online database, Cbioportal (https://www.cbioportal.org). The results demonstrated that RNF146 genetic alterations were not significant in GC and CRC (Figure 2A). However, data from eight pairs of fresh GC samples and 42 pairs of fresh CRC samples showed that the expression of RNF146 mRNA was higher in GIC tissues than in adjacent noncancerous tissues (Figure 2B,C), suggesting that overexpression of RNF146 protein may be regulated at the post‐transcriptional level in GIC. Furthermore, based on bioinformatic analysis tools (TargetScan; www.targetscan.org and miRanda; www.microrna.org), we speculated that miR‐3133 might be a putative miRNA that regulates RNF146 (Figure 2D). KEGG pathway analyses revealed that miR‐3133 may have a vital function in pathways involved in cancer, Hippo signalling, etc. (Figure 2E). Fluorescence in situ hybridization (FISH) assays were performed to verify the expression of miR‐3133 in tumour tissues, and the results showed that the expression was decreased in the cytoplasm of fresh GIC tissues (Figure 2F). Next, we aimed to clarify whether miR‐3133 and RNF146 mRNA can bind to each other. After transfection of miR‐3133 mimics and inhibitors into HGC‐27 and HCT‐116 cells, we found that miR‐3133 mimics markedly reduced the mRNA and protein expression of RNF146, whereas opposite trends were observed after transfection with miR‐3133 inhibitor (Figure 2G,H). In addition, wild‐type (WT) and mutant (Mut) miR‐3133 were constructed (Figure 2D) and dual‐luciferase assays were performed. miR‐3133 mimics decreased, but miR‐3133 inhibitors remarkably enhanced the luciferase activity of RNF146 in both transfected HGC‐27 and HCT‐116 cells (Figure 2I,J). Taken together, our data elucidated that RNF146 is a target of miR‐3133 in GIC.

### 3.3 miR‐3133 inhibits proliferation and chemoresistance in GIC

**FIGURE 3:**
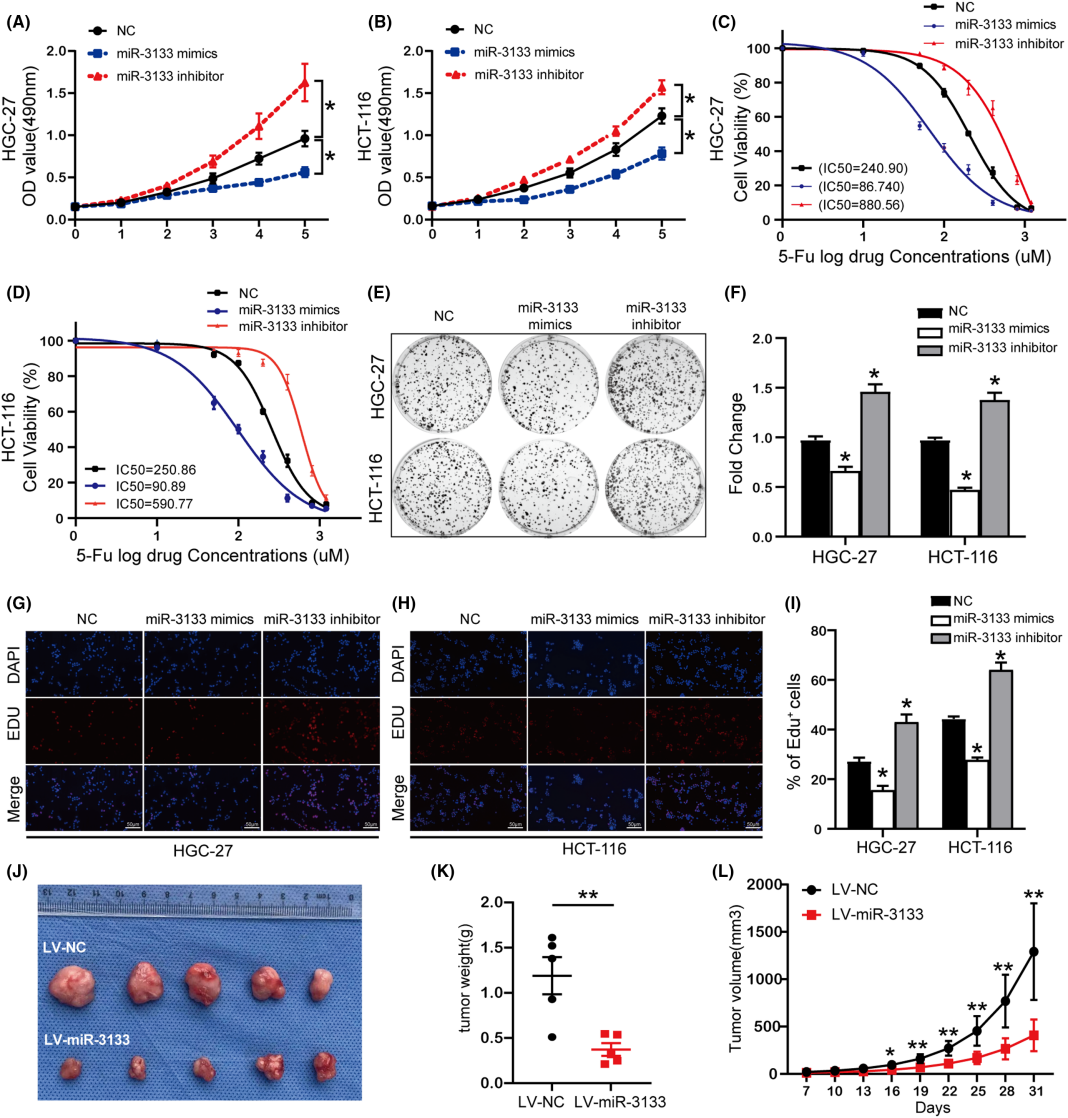
miR‐3133 inhibits cell proliferation and chemoresistance in gastrointestinal cancer (GIC). (A)–(I) Cells were transfected with miR‐3133 mimics, miR‐3133 inhibitor and negative control and subjected to the CCK‐8 assay (A) and (B), drug sensitivity experiments (C) and (D), colony formation assay (E) and (F) and EdU incorporation assay (G)–(I). (J)–(L) Xenograft tumour model derived from HGC‐27 cells expressing LV‐NC or LV‐miR‐3133. (J) Images of xenograft tumours harvested at the end of experiments; (K) Comparison of the average weight of collected tumours from the above two groups; (L) Growth curves of xenograft tumours (*p < 0.05, **p < 0.01 vs. corresponding control groups).

As miR‐3133 was found to be a negative regulator of RNF146, we hypothesized that miR‐3133 may act as a tumour‐suppressive miRNA in tumorigenesis and progression. To test this hypothesis, HGC‐27 and HCT‐116 cells were transfected with miR‐3133 mimics, miR‐3133 inhibitor or negative control. The results of CCK‐8 assay revealed that the upregulation of miR‐3133 suppressed the proliferative capacity of HGC‐27 and HCT‐116 cells, whereas the miR‐3133 inhibitor showed the opposite effect (Figure 3A,B). In line with the above results, the colony formation assay and EdU assay further validated that the expression of miR‐3133 truly affects the proliferation of cancer cells (Figure 3E–I). In addition, the IC_50_ value of cancer cells decreased or increased significantly after transfection with miR‐3133 mimics or miR‐3133 inhibitors, respectively, indicating that miR‐3133 affected the sensitivity of cancer cells to 5‐FU treatment (Figure 3C,D). Furthermore, we determined the role of miR‐3133 in the growth of GC xenografts in nude mice. We subcutaneously injected HGC‐27 cells, with or without overexpressed miR‐3133, into nude mice and recorded tumour formation and growth for 31 days. As expected, the weight of xenografts from lentiviral‐miR‐3133 mice grew at a slower rate compared to that in the control group (Figure 3J–L). Collectively, these data indicate that miR‐3133 suppresses the proliferation and chemoresistance of cancer cells.

### 3.4 miR‐3133 activates the Hippo‐YAP1 pathway

**FIGURE 4:**
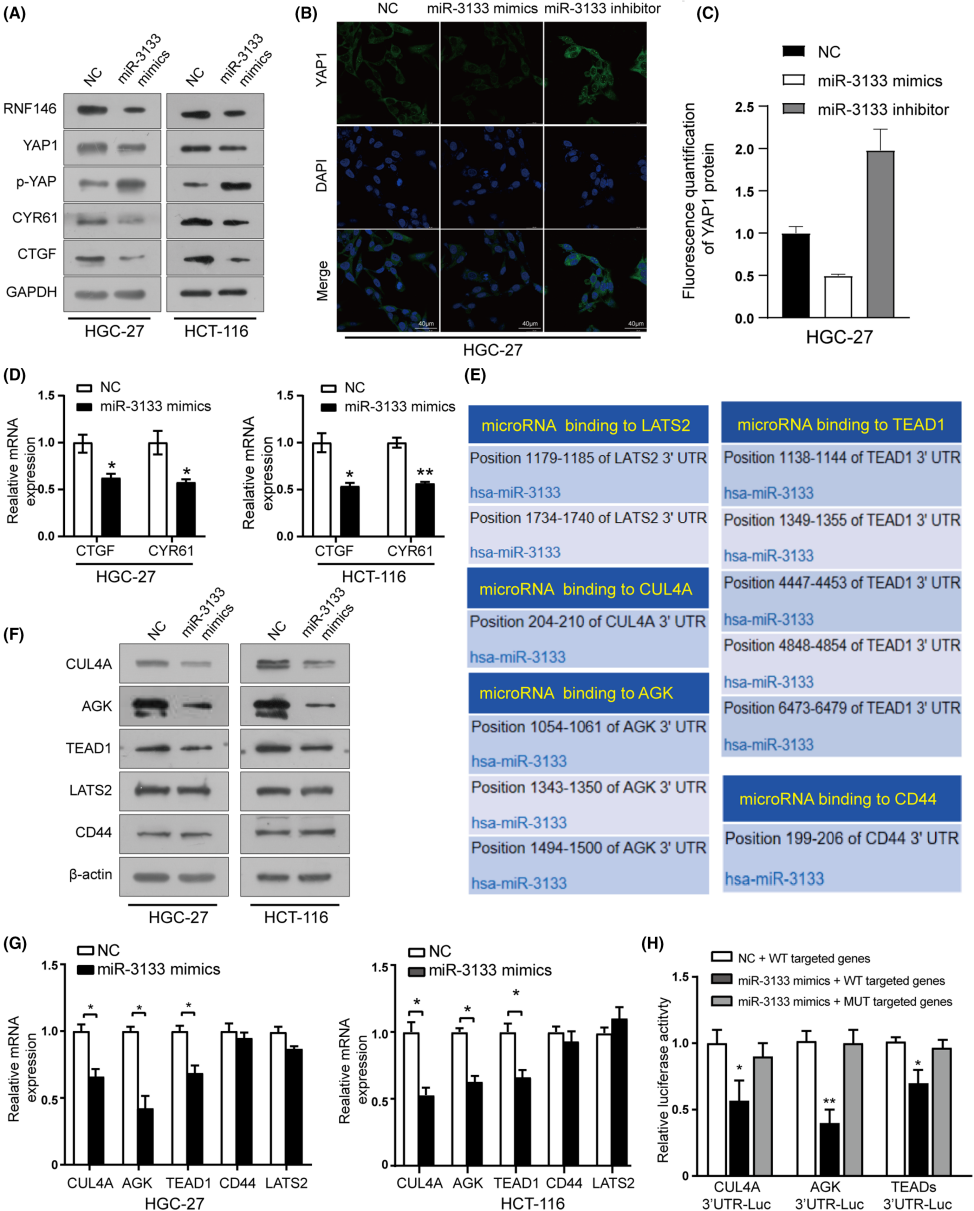
miR‐3133 activates the Hippo‐YAP1 pathway. (A) The levels of YAP1, p‐YAP and their downstream target genes in HGC‐27 cells and HCT‐116 cells transfected with miR‐3133 mimics were analysed using western blotting. (B) and (C) Immunofluorescence staining and quantitation of fluorescence intensity of YAP1 in HGC‐27 cells after transfection with miR‐3133 mimics and miR‐3133 inhibitor. (D) RT‐qPCR results for CTGF and CYR61 in HGC‐27 and HCT‐116 cells transfected with miR‐3133 mimics. (E) Predicted miR‐3133 target sequences in the 3′‐UTRs of CUL4A, AGK, CD44, LATS2 and TEAD1 by TargetScan website (www.targetscan.org). (F) and (G) Western blot results and RT‐qPCR results of CUl4A, AGK, CD44, LATS2 and TEAD1 expression levels in HGC‐27 and HCT‐116 cells transfected with miR‐3133 mimics or negative control. (H) Luciferase activity of CUL4A 3′‐UTR, AGK 3′‐UTR TEADs 3′‐UTR and their mutants were determined in HGC‐27 cells transfected with miR‐3133 mimics or negative control (*p < 0.05, **p < 0.01 vs. corresponding control groups).

As our data showed that RNF146 affects the expression of YAP1 and downstream target genes, we speculated that miR‐3133 could affect the Hippo‐YAP1 pathway by targeting RNF146. As expected, overexpression of miR‐3133 reduced RNF146 and total YAP1 protein levels but increased p‐YAP protein levels in HGC‐27 and HCT‐116 cells (Figure 4A), and two related target genes CTGF and CYR61 were concomitantly decreased (Figure 4A,D). Immunofluorescence results confirmed that YAP1 was reduced as well as increased after transfection with miR‐3133 mimics and inhibitors, respectively (Figure 4B,C; Figure S2A). Taken together, miR‐3133 activated the Hippo pathway in GC and CRC cells.

Notably, the restoration of RNF146 only partially abrogated miR‐3133‐induced suppression of GC cell proliferation (Figure S2B), suggesting that, in addition to RNF146, other targets of miR‐3133, to date unidentified, may also be involved. Based on the bioinformatic analysis, we found that certain Hippo pathway‐related genes, such as CUL4A, AGK, CD44, LATS2 and TEAD1 are also potential targets of miR‐3133 (Figure 4E). To further validate these results, RT‐qPCR and western blotting assays were performed, and the results showed that upregulation of miR‐3133 reduced the protein and mRNA expression levels of CUL4A, AGK and TEAD1, but not those of CD44 and LATS2 (Figure 4F,G). The luciferase reporter assay results indicated that upregulation of miR‐3133 significantly repressed the luciferase activity of vectors carrying the WT 3’‐UTR of CUL4A, AGK and TEAD1, but not the mutant (Figure 4H). Overall, these results indicated that miR‐3133 activates the Hippo pathway by targeting RNF146, CUL4A, AGK and TEAD1.

### 3.5 miR‐3133 activates the p53 signalling pathway

**FIGURE 5:**
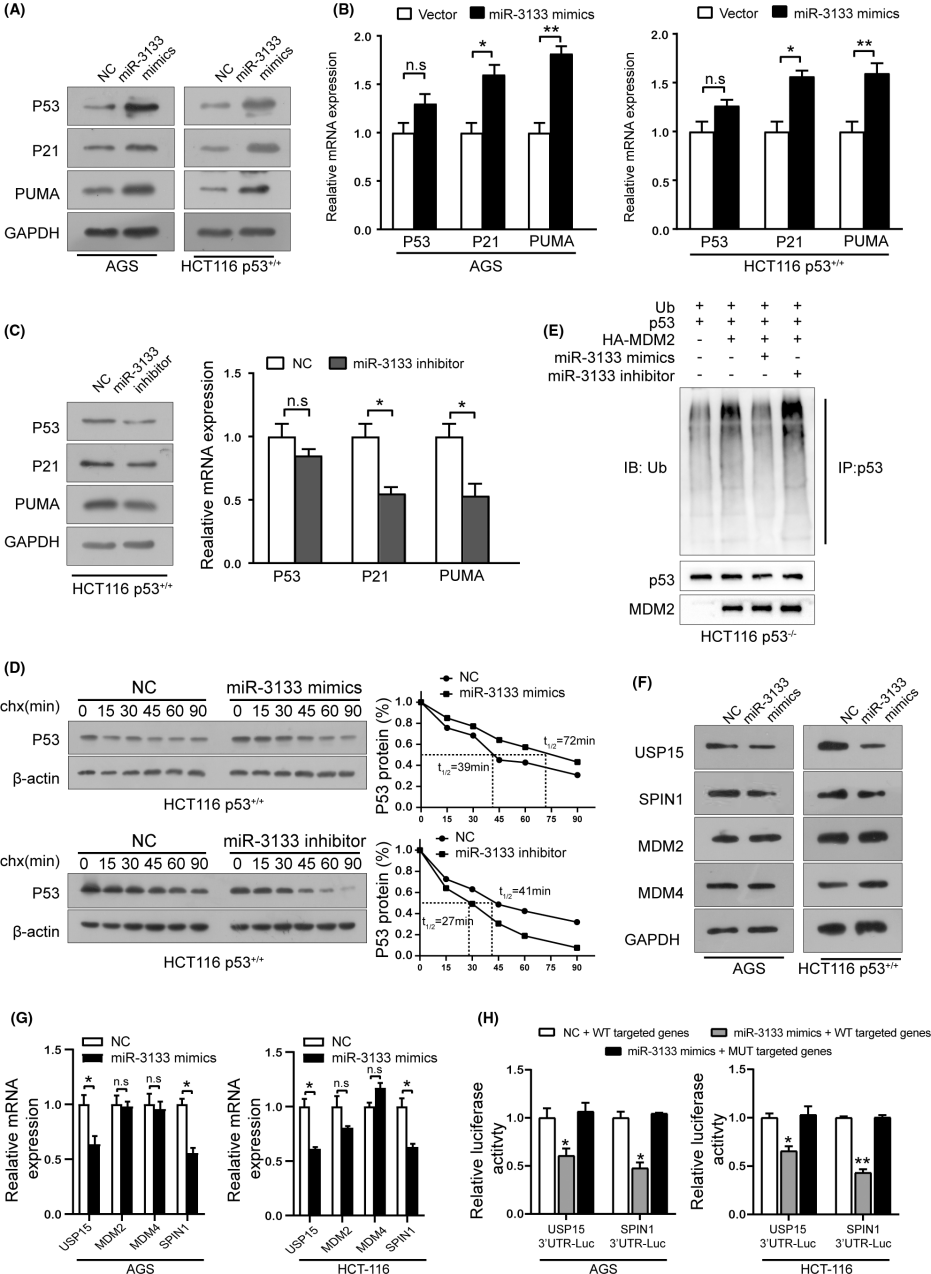
miR‐3133 activates the p53 signalling pathway. (A) and (B) Western blotting and RT‐qPCR results of p53, p21 and PUMA expression levels in AGS and HCT‐116 p53^+/+^ cells transfected with miR‐3133 mimics. (C) Western blot and RT‐qPCR results of p53, p21 and PUMA expression levels in HCT116 p53^+/+^ cells transfected with the miR‐3133 inhibitor. (D) The half‐life of p53 was detected after transfection with miR‐3133 mimics or miR‐3133 inhibitor, treated with 50 μg/mL of cycloheximide (CHX), and harvested at different time points, as indicated. (E) MDM2‐induced p53 ubiquitination in HCT116 p53^−/−^ cells transfected with the indicated constructions for 36 h and treated with MG132 for 6 h before being harvested was examined in an in vivo ubiquitination assay. (F) and (G) Western blot and RT‐qPCR results of USP15, MDM2, MDM4 and SPIN1 expression levels in AGS cells and HCT116 p53^+/+^ transfected with miR‐3133 mimics or negative control. (H) Luciferase activity of USP15 3′‐UTR, SPIN1 3′‐UTR and their mutants in AGS and HCT‐116 cells transfected with miR‐3133 mimics and negative control (*p < 0.05, ** *p* < 0.01 vs. corresponding control groups).

The above data suggested that CUL4A was regulated by miR‐3133; however, a previous study showed that CUL4A physically associates with MDM2 and participates in MDM2‐mediated proteolysis of p53.^19^ Therefore, we conducted a series of experiments to explore whether miR‐3133 regulates the p53 signalling pathway. Interestingly, as shown in Figure 5A,B, upregulation of miR‐3133 dramatically elevated p53 protein levels in AGS (expressing wild‐type p53) and HCT‐116 (p53^+/+^) cells without affecting mRNA levels. Both protein and mRNA levels of p53 target genes such as p21 and PUMA were increased in miR‐3133‐overexpressing cells. In contrast, opposite effects were observed in HCT116 p53^+/+^ cells after transfection with the miR‐3133 inhibitor (Figure 5C). To further clarify the effect of miR‐3133 on p53, HCT116 p53^+/+^ cells were treated with cycloheximide (CHX) and harvested in a time‐dependent manner. A dramatic increase in the stability and longer half‐life of p53 was observed in miR‐3133‐overexpressing cells, whereas a shorter half‐life and decreased p53 activity were observed in miR‐3133‐knockdown cells (Figure 5D). In addition, we performed ubiquitination assays to investigate whether miR‐3133 regulates p53 ubiquitination in HCT116 p53^−/−^ cells. As expected, MDM2 induced p53 ubiquitination, which was relieved by miR‐3133 mimics or further enhanced by the miR‐3133 inhibitor (Figure 5E). These results suggest that miR‐3133 stabilizes the p53 protein by inhibiting MDM2‐mediated ubiquitination.

Notably, bioinformatics analysis results showed that, in addition to CUL4A, miR‐3133 could also bind USP15, MDM2, MDM4 and SPIN1 (Figure S2C), which have previously been reported to be involved in the regulation of the p53 pathway.^18^, ^20^, ^33^ We then investigated the expression of these genes in miR‐3133‐upregulated or ‐downregulated cells by western blotting and RT‐qPCR and found that only the expression of USP15 and SPIN1 was reduced in miR‐3133‐transfected cells but increased in miR‐3133 antagomir‐transfected cells (Figure 5F,G). Luciferase reporter assays further demonstrated that miR‐3133 mimics markedly decreased the luciferase activity of SPIN1 and USP15 3′‐UTR plasmids in AGS and HCT‐116 cells, but not that of SPIN1 and USP15 Mut‐3′‐UTR (Figure 5H). Taken together, miR‐3133 activates the p53 signalling pathway by targeting CUL4A, USP15 and SPIN1.

### 3.6 Low expression of miR‐3133 induced by promoter methylation is associated with poor prognosis in patients

**FIGURE 6:**
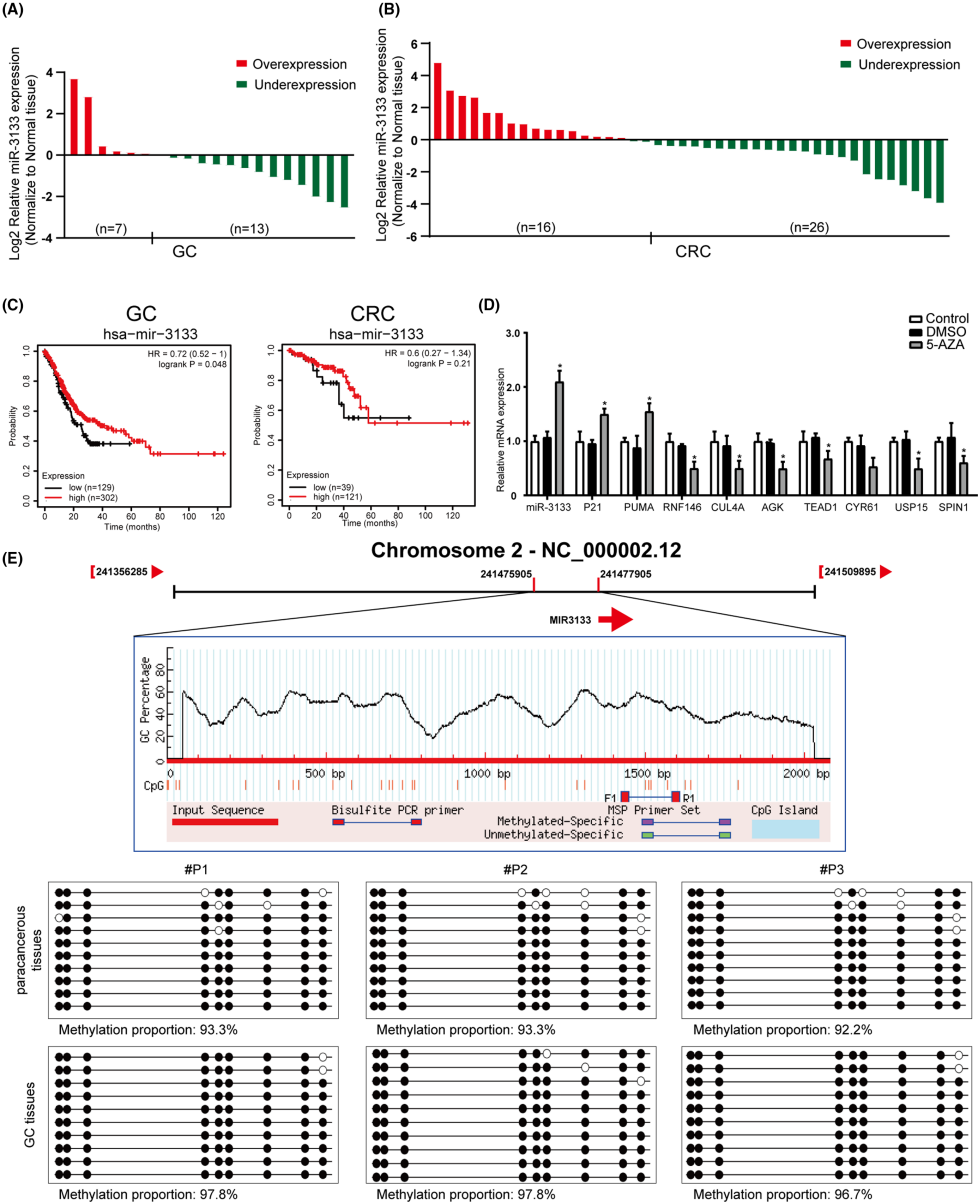
Low expression of miR‐3133 induced by promoter methylation is associated with poor prognosis in patients. (A) and (B) miR‐3133 was detected by RT‐qPCR in 20 pairs of gastric cancer (GC) tissues, 42 pairs of colorectal cancer (CRC) tissues and adjacent normal tissues. (C) Overall survival curve of miR‐3133 in GC and CRC from Kaplan–Meier plotter analysis (https://kmplot.com/analysis/). (D) Levels of miR‐3133, p21, PUMA, RNF146, CUL4A, AGK, TEAD1, CYR61, USP15 and SPIN1 were assayed after treatment with or without 5‐aza‐2′‐deoxycytidine (5‐Aza‐dC), an inhibitor of DNA methyltransferase, for 48 h in AGS cells. (E) DNA methylation level of the miR‐3133 CpG island region in GC tissues and noncancerous gastric tissues analysed by bisulfite sequencing PCR (*p < 0.05, **p < 0.01 vs. corresponding control groups).

The above data indicated that miR‐3133 was expressed at lower levels in tumour tissues (Figure 2F). To further verify these conclusions, RT‐qPCR was performed on 20 pairs of fresh GC tissues and 42 pairs of fresh CRC tissues. As shown in Figure 6A,B, miR‐3133 expression was significantly decreased in 13/20 (65%) GC and 26/42(61.9%) CRC tissues. In addition, the clinicopathological characteristics of the 42 patients were collected to explore their relationship with miR‐3133 expression. As shown in Table 2, miR‐3133 expression was strongly correlated with the depth of tumour size (*p* = 0.023), and Kaplan–Meier analysis (https://kmplot.com/) indicated that GC patients with low miR‐3133 expression levels had worse overall survival than those with high miR‐3133 expression levels, a phenomenon not seen in CRC patients (Figure 6C). Hypermethylation of DNA‐induced epigenetic silencing of miRNAs is the primary mechanism for cancer progression.^34^ Therefore, we designed specific primers to conduct the BSP assay using six pairs of GC tissues. The results showed that the methylation level of CpG sites in the miR‐3133 promoter was higher in tumour tissues than in adjacent normal tissues (97.433% ± 0.2319% vs. 92.9333% ± 0.2319%, *p* < 0.001) (Figure 6E; Figure S2D). AGS cells were treated with 5‐aza‐dC (5‐AZA, a DNA methyltransferase inhibitor) to verify whether miR‐3133 expression is regulated by DNA methylation. As shown in Figure 6D, miR‐3133 expression was significantly upregulated after 5‐Aza treatment. Its target molecules, RNF146, AGK, CUL4A, TEAD1, USP15 and SPIN1, decreased concomitantly, and the key effectors of the Hippo pathway, CYR61, and the p53 downstream targets, p21 and PUMA, were altered accordingly. Collectively, our data indicated that low expression of miR‐3133 is associated with tumour progression, and promoter methylation was identified as the cause of its reduced expression.

**TABLE 2:** The association between miR‐3133 expression and clinicopathological factors in patients with colorectal cancer.

Clinicopathological factors	*N*	miR‐3133 expression (%)		*p*‐Value
		Low	High	
Age (years)	42	26 (61.9)	16 (38.1)	
≤55	17	9 (52.9)	8 (47.1)	0.324
>55	25	17 (68.0)	8 (32.0)	
Sex				
Female	15	8 (53.3)	7 (46.7)	0.394
Male	27	18 (66.7)	9 (33.3)	
Tumour size (cm)				
≤5	17	7 (41.2)	10 (58.8)	**0.023**
>5	25	19 (76.0)	6 (24.0)	
Lymph node metastasis				
Negative	26	14 (53.8)	12 (46.2)	0.206
Positive	16	12 (75.0)	4 (25.0)	
TNM stage				
I‐II	24	12 (50.0)	12 (50.0)	0.109
III‐IV	18	14 (77.8)	4 (22.2)	
Differentiation				
Well‐middle	26	15 (57.7)	11 (42.3)	0.474
Poorly	16	11 (68.8)	5 (31.2)	

*Note*: All the data were analysed by the chi‐square test or Fisher exact test. Entries in bold font indicate statistically significant (*p* < 0.05).

### 3.7 miR‐3133 affects cancer progression through multi‐targets

**FIGURE 7:**
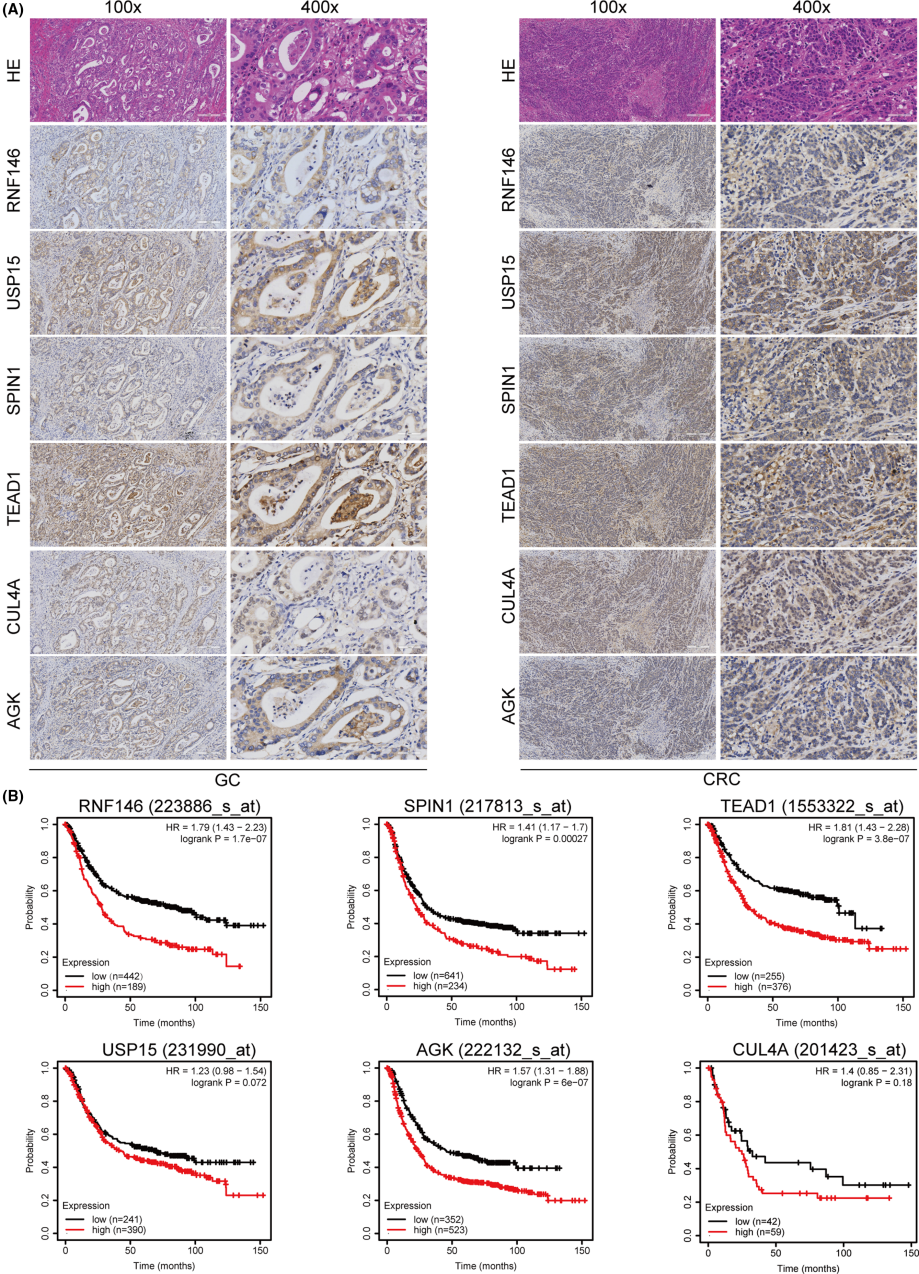
miR‐3133 affects cancer progression through multiple targets. (A) Immunohistochemical staining of RNF146, USP15, SPIN1, TEAD1, CUL4A and AGK in gastric cancer (GC) and colorectal cancer (CRC) tissues with low miR‐3133 expression. (B) The prognostic roles of RNF146, CUL4A, AGK, TEAD1, YAP1, USP15 and SPIN1 in GC were determined by Kaplan–Meier analysis (https://kmplot.com/analysis/).

The above data indicate that miR‐3133 regulates the Hippo and p53 signalling pathways through multiple targets in GC and CRC cells. To further verify the relationship between miR‐3133 and these targets, we used immunohistochemistry to detect the expression of target molecules in paraffin‐embedded samples from 20 patients with GC and 42 patients with CRC. As shown in Figure 7A, in the tissues with low expression of miR‐3133, the related target molecules were highly expressed. We then evaluated the IHC staining scores of the related target molecules and analysed their correlation with miR‐3133. The results showed that the expression of miR‐3133 was negatively correlated with SPIN1 (*p* = 0.008), TEAD1 (*p* = 0.016) and RNF146 (*p* = 0.044) (Table 3). Furthermore, the prognostic roles of RNF146, CUL4A, AGK, TEAD1, USP15 and SPIN1 in GC were evaluated using the KM plotter website (Figure 7B). The results showed that high expression of RNF146, TEAD1, YAP1 and SPIN1 indicated poor prognosis in GC and CRC. Overall, these results indicate that miR‐3133 affects cancer progression through multiple targets.

**TABLE 3:** The correlation of miR‐3133 and its targets.

	miR‐3133 expression		*χ* ^2^	*p*‐Value
	Low	High		
CUL4A				
Low	8	9	2.669	0.102
High	18	7		
USP15				
Low	9	10	3.109	0.078
High	17	6		
SPIN1				
Low	7	11	7.076	0.008
High	19	5		
AGK				
Low	9	10	3.109	0.078
High	17	6		
TEAD1				
Low	8	11	5.768	0.016
High	18	5		
RNF146				
Low	8	10	4.072	0.044
High	18	6		

## 4 DISCUSSION

Our study showed that the expression of RNF146 was elevated in GC and that its overexpression was associated with worse survival outcomes and more adverse clinicopathological features of GC. Depletion of RNF146 reduced the proliferative ability of GIC cells by activating the Hippo pathway in vivo and in vitro. We also found that miR‐3133, as an upstream regulator of RNF146, plays a tumour‐suppressive role in GIC. miR‐3133 was poorly expressed in GC and CRC tissues and negatively correlated with patient clinical features. Mechanistically, we found that miR‐3133 negatively regulates multiple targets, including RNF146, AGK, CUL4A, SPIN1, USP15 and TEAD1, thereby affecting the activity of the Hippo and p53 signalling pathways. However, miR‐3133 is epigenetically regulated via DNA promoter methylation. Thus, this study not only provides novel insights into the upstream regulatory network of the Hippo and p53 signalling pathways but also provides the rationale for miR‐3133 as a promising therapeutic target for GIC.

Abnormalities in signalling pathways, the hallmark of cancer, are frequently caused by the dysregulation of one or more molecules in these pathways.^35^ Uncontrolled cell proliferation is the main obstacle to cancer treatment, and both the Hippo and p53 signalling pathways are essential for cancer cell growth and chemoresistance.^33^, ^36^ Using bioinformatics prediction, we found that miR‐3133 can target and regulate the expression of some proteins that are known to affect the Hippo and p53 pathways as previous studies. For instance, CUL4A mediates the ubiquitination of LATS1 to regulate YAP activity in hepatocellular carcinoma,^37^ AGK inhibits the activation of the Hippo pathway proteins in GC^38^ and CD44 functions upstream of the Hippo signalling pathway and attenuates its activation in glioblastoma.^39^ Some proteins, such as MDM2, MDM4 and TEAD1, are the key components of the Hippo and p53 signalling pathways. Based on bioinformatic predictions, we further screened the target genes regulated by miR‐3133 through experimental verification. Our data suggest that the upregulation of miR‐3133 can downregulate these target genes and lead to the inactivation of YAP and decreased ubiquitination‐mediated p53 degradation, thus changing the expression of downstream target genes and reducing the proliferation and drug resistance of tumour cells. This pattern of multiple targets co‐regulating signalling pathways provides a novel understanding of miRNA regulating signal transduction.

RNF146 is a target gene of miR‐3133. Several studies have shown that RNF146 can promote the progression of various cancers by activating the Wnt signalling pathway.^25^, ^26^, ^40^ In addition, Wang et al. found that RNF146 could mediate NF2 regulation of the Hippo pathway,^41^ whereas Cao et al. showed that RNF146 is involved in the formation of the ubiquitin complex (Slug/GSK3β/RNF146) and induction of metastasis of GC.^42^ However, the expression of RNF146 in GC and its upstream and downstream regulatory mechanisms remain unclear. Here, we verified for the first time that the expression of RNF146 is upregulated in GC and that the downregulation of RNF146 leads to the activation of the Hippo pathway and reduction of the proliferative ability of GC and CRC cells.

Recently, the role of miRNAs in tumorigenesis has been validated by numerous studies. miRNAs may modulate tumorigenesis, proliferation, apoptosis and chemoresistance of cancers through multiple signalling pathways.^43^, ^44^ In this study, we found that a newly discovered miRNA, named miR‐3133, is involved in the proliferation of GIC cells. To date, the role of miR‐3133 in cancer has been poorly investigated, except in retinoblastoma and clear cell renal cell carcinoma.^45^, ^46^ In line with the above findings, our data indicate that miR‐3133 plays a tumour‐suppressive role in GIC. Previous studies have shown that miR‐3133 is expressed at low levels in tumour tissues, but the mechanism of its downregulation remained unclear.^45^, ^46^ Epigenetic mechanisms can alter gene expression patterns and genomic stability without altering DNA sequences, including DNA methylation, histone modification, chromatin remodelling and altered expression of noncoding RNAs.^47^ DNA methylation is an important part of the epigenetic regulatory mechanism, which refers to the process by which the C‐5 position of the cytosine of a nucleotide is covalently modified to 5‐ methylation cytosine (5mC) by a methyl supplied by S‐adenosine methionine through DNA methyltransferase.^48^ In this paper, methylation sequencing was used to verify the high methylation level of CpG islands in the miR‐3133 promoter. Methylation inhibitors were used to restore the expression of miR‐3133 in tumour cells, confirming that promoter methylation of miR‐3133 was the cause of its low expression. Therefore, we explain that miR‐3133 is expressed at low levels in GICs due to methylation, further improving the multi‐target and dual‐pathway regulatory network centred on miR‐3133. A limitation of our study was that CRC patients with high miR‐3133 did not show benefit with OS outcomes (*p* = 0.21, *N* = 160, Figure 6C), which may be attributed to the small sample size and/or the rectal location of the tumour in most of these patients.

In summary, our data indicate that miR‐3133 inhibits GC and CRC cell proliferation by activating the Hippo and p53 signalling pathways via multiple targets. Methylation of the miR‐3133 promoter in tumour tissues downregulated miR‐3133 expression. Therefore, this research suggested that miR‐3133 may function as a potential target in anti‐GC and ‐CRC treatment and provided a basis for further comprehensive follow‐up studies.

## AUTHOR CONTRIBUTIONS

**Xiaojun Xiang:** Conceptualization (lead); data curation (equal); funding acquisition (lead); writing – review and editing (lead). **Ling Zhou:** Investigation (lead); writing – original draft (lead). **Hui Guo:** Investigation (lead). **Quan Liao:** Data curation (lead); methodology (lead). **Jianping Zou:** Investigation (supporting). **Yi Le:** Formal analysis (supporting); investigation (supporting). **Ziling Fang:** Formal analysis (supporting); supervision (supporting); validation (supporting); writing – review and editing (supporting). **Jianping Xiong:** Resources (equal); supervision (equal); writing – review and editing (equal). **Shanshan Huang:** Conceptualization (equal); data curation (supporting); formal analysis (supporting); funding acquisition (equal); project administration (equal); writing – review and editing (equal). **Jun Deng:** Funding acquisition (equal); methodology (equal); supervision (equal); writing – review and editing (equal).

## FUNDING INFORMATION

The authors thank the Human Genetic Resources Center and Medical Innovation Center of the First Affiliated Hospital of Nanchang University and for their support. Our study was funded in part by the Jiangxi Provincial Outstanding Young Talents projects (2019BCB23020), Key Research and Development Programs of Jiangxi Province (20202BBGL73055), Natural Science Foundation of China (82160464, 82260491,82202869, and 82260571), Natural Science Foundation of Jiangxi Province (20192ACB20028), Jiangxi Key Laboratory for Individualized Cancer Therapy (20202BCD42011), Jiangxi Provincial Young Talents projects (20204BCJ23016) and Special fund for innovation of Postgraduates in Jiangxi Province (YC2022—B055).

## CONFLICT OF INTEREST STATEMENT

The authors declare no conflict of interest.

## Supporting information


Data S1:

## Data Availability

The datasets used and analysed during the current study are available from the corresponding authors (Xiaojun Xiang et al.) upon reasonable request.
