# Supplementary material for: miR‐3133 inhibits gastrointestinal cancer progression through activation of Hippo and p53 signalling pathways via multi‐targets
Source: J Cell Mol Med. 2023 Aug 9;27(20):3090–106. doi: 10.1111/jcmm.17880 (PMC10568676; doi:10.1111/jcmm.17880)
Supplement: Supplementary file 1 — Data S1: [file JCMM-27-3090-s001.docx]

Supplementary Material

­­miR-3133 inhibits gastrointestinal cancer progression through activation of Hippo and p53 signaling pathways via multi-targets

# Supplementary Figures and Tables

##
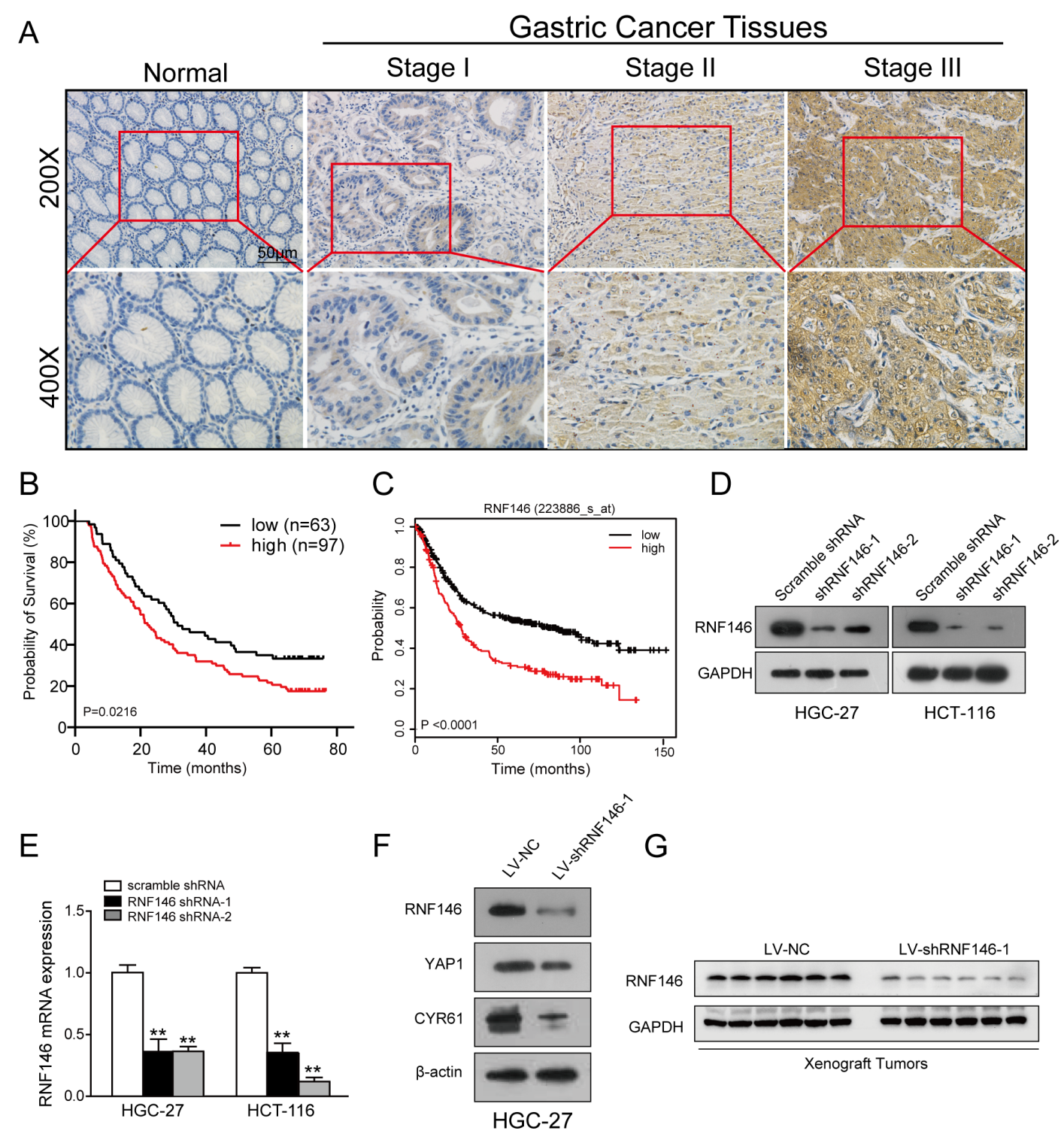
Supplementary Figures

## Supplementary FIGURE 1. RNF146 acts as an oncogene in GIC. (A) Representative IHC images of RNF146 staining in GC tissues. (B) Patients with low RNF146 expression levels showed longer survival times (*p*=0.0216, log-rank test). (C) The prognostic role of RNF146 in GC was determined by extracting data from the Kaplan–Meier website. (D-E) Knock-down effects of RNF146 in GIC cells were detected by western blotting and RT-qPCR. (F) RNF146, YAP1, and CTGF protein expression from HGC-27 cells expressing LV-scramble-shRNA (LV-NC) or LV-shRNF146. (G) Knock-down effects of RNF146 protein were confirmed in Xenograft tumors.

**
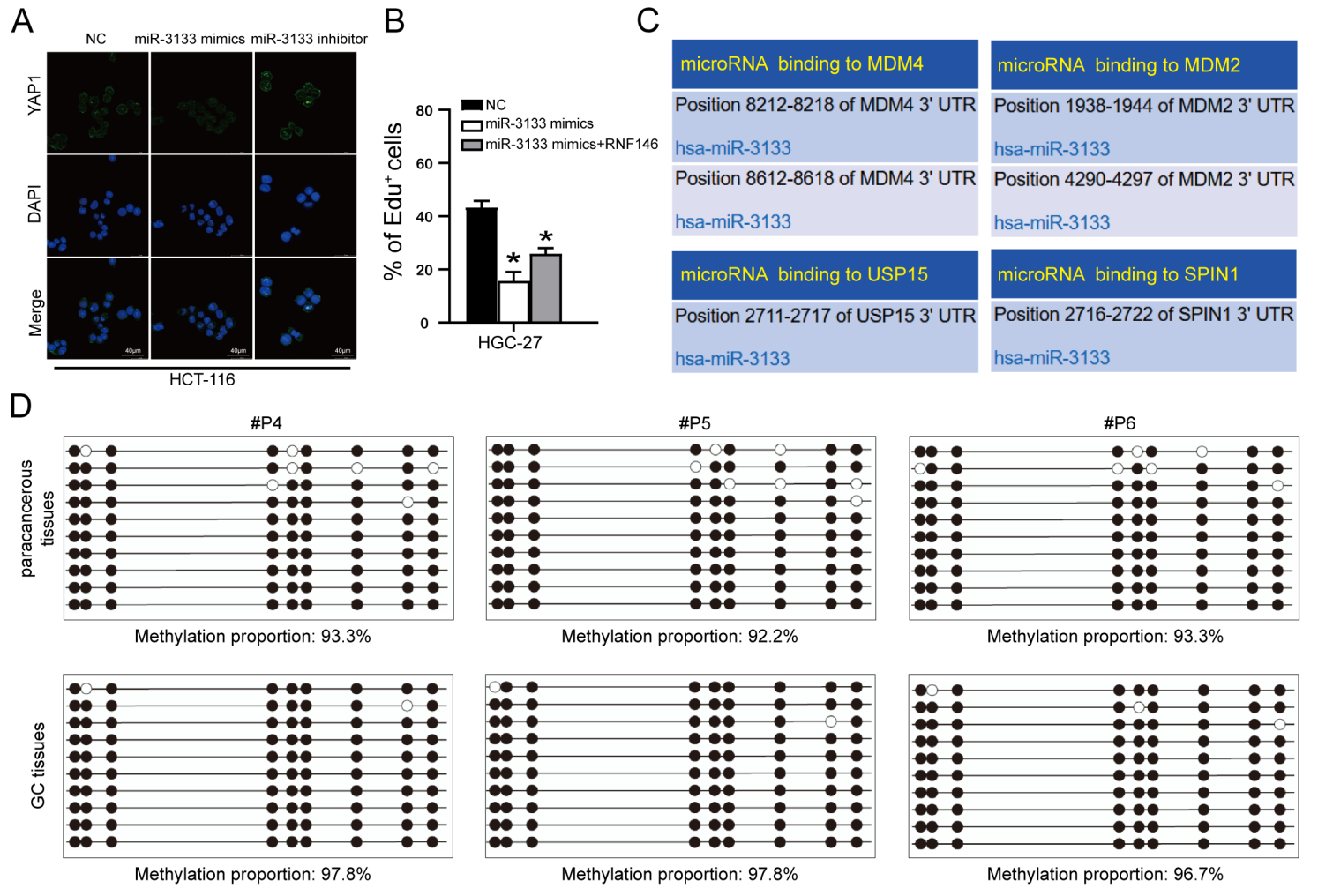
**

**Supplementary FIGURE 2.** The roles of miR-3133: supplementary for the formal figures. (A) Immunofluorescence staining of YAP1 in HCT-116 cells after transfection with miR-3133 mimic and miR-3133 inhibitor. (B) EdU incorporation assay in HGC-27 cells transfected with the indicated constructs. (C) Predicted miR-3133 target sequences in the 3′-UTRs of MDM4, MDM2, USP15, and SPIN1. (D) DNA methylation level of the miR-3133 CpG island region in GC tissues and noncancerous gastric tissues analyzed by BSP. (**p* < 0.05, ***p* < 0.01 vs. corresponding control groups)

## Supplementary tables

**Supplementary Table 1. Primer sequences used in qRT-PCR assays.**

| **Gene** | **Primer sequences** |
| --- | --- |
| miR-3133 | Stem-loop: 5'-GTCGTATCCAGTGCAGGGTCCGAGGTATTCGCACTGGATACGACATTGGG-3' |
|  | Forward: 5'-CGCGCGTAAAGAACTCTTAAAA-3' |
|  | Reverse: 5'-AGTGCAGGGTCCGAGGTATT-3' |
| RNF146 | Forward: 5'-GGACGTCGCAGGAAGATTAAG-3' |
|  | Reverse: 5'-CAATGGAGGTGTCTGGTGCT-3' |
| TEAD1 | Forward: 5'-GATGATGCTGGGGCTTTTTA-3' |
|  | Reverse: 5'-GCCATTCTCAAACCTTGCAT-3' |
| CYR61 | Forward: 5'-CAGGACTGTGAAGATGCGGT-3' |
|  | Reverse: 5'-GCCTGTAGAAGGGAAACGCT-3' |
| CUL4A | Forward: 5'-CAGCGGCTCTGATTACAGACCTCG-3' |
|  | Reverse: 5'-GTCTTCACAGGCCTGACGCAGT-3' |
| CTGF | Forward: 5'-CCTGGTCCAGACCACAGAGT-3′ |
|  | Reverse: 5′-TGGAGATTTTGGGAGTACGG-3′ |
| AGK | Forward: 5'-CCTGACACCATCAGCAAAGG-3' |
|  | Reverse: 5'-CTCCGGGATAAGCAAAGTGC-3' |
| CD44 | Forward: 5′- AGCAGCGGCTCCACCATCGAGA-3′ |
|  | Reverse: 5′- TCGGATCCATGAGTCACAGTG-3′ |
| LATS2 | Forward: 5'-ACATTCACTGGTGGGGACTC-3' |
|  | Reverse: 5'-GTGGGAGTAGGTGCCAAAAA-3' |
| P53 | Forward: 5'-CCCAAGCAATGGATGATTTTGA-3' |
|  | Reverse: 5'-GGCATTCTGGGAGCTTCATCT-3' |
| P21 | Forward: 5'-CTGGACTGTTTTCTCTCGGCTC-3' |
|  | Reverse: 5'-TGTATATTCAGCATTGTGGGAGGA-3' |
| PUMA | Forward: 5'-ACAGTACGAGCGGCGGAGACAA-3' |
|  | Reverse: 5'-GGCGGGTGCAGGCACCTAATT-3' |
| USP15 | Forward: 5'-AGGGGACACCTGGTATCTAGT-3' |
|  | Reverse: 5'-CATGTTCCCGTTCTCACAGAG-3' |
| MDM2 | Forward: 5'-TTAGAGCACCCTGTCACCAC-3' |
|  | Reverse: 5'-ATGTAATTCAGCATCCACCC-3' |
| MDM4 | Forward: 5'-TGTGGTGGAGATCTTTTGGG-3' |
|  | Reverse: 5'- GCAGTGTGGGGATATCGT-3' |
| SPIN1 | Forward: 5'-CAGAGCTGATGCAGGCCAT-3' |
|  | Reverse: 5'-ACTGGGTAACAGGGCCATTG-3' |
| GAPDH | Forward: 5'-CACCCACTCCTCCACCTTTG-3' |
|  | Reverse: 5'-CCACCACCCTGTTGCTGTAG-3' |

**Supplementary Table 2. Primer sequences used in qRT-PCR assays.**

| primary antibodies | | details |
| --- | --- | --- |
| RNF146 | 1:1000, HPA027209, Atlas, Switzerland；1：500，bs-11669R, Bioss，Beijing, China | |
| YAP1 | 1:1000, #14074, Cell Signaling Technology, MA, USA | |
| P-YAP1 | 1:1000, #13008, Cell Signaling Technology, MA, USA | |
| CTGF | 1:1000, ab6992, Abcam, Cambridge, USA | |
| CYR61 | 1:1000, #14479, Cell Signaling Technology, MA, USA | |
| CUL4A | 1:1000, 14851-1-AP, Proteintech, Wuhan, China | |
| AGK | 1:1000, GTX107413, GeneTex, Texas, USA | |
| TEAD1 | 1:1000, 13283-1-AP, Proteintech, Wuhan, China | |
| LATS2 | 1:1000, #5888, Cell Signaling Technology, MA, USA | |
| CD44 | 1:1000, 60224-1-Ig, Proteintech, Wuhan, China | |
| p53 | 1:1000, 60283-2-Ig, Proteintech, Wuhan, China | |
| MDM2 | 1:1000, ab16895, Abcam, Cambridge, USA | |
| MDM4 | 1:1000, 17914-1-AP, Proteintech, Wuhan, China | |
| p21 | 1:1000, ab109199, Abcam, Shanghai, China | |
| PUMA | 1:1000, ab9643, Abcam, Cambridge, USA | |
| SPIN1 | | 1:1500; #12,105-1-AP, Proteintech, Wuhan, China) |
| USP15 | | 1:2000, #66310; Cell Signaling Technology, Danvers, USA |
| GAPDH | | 1:15000, Proteintech, Wuhan, China |
| β-actin | | 1:2000; #AF7018; Affinity, Jiangsu, China |
